# Factors influencing the delivery of cancer pathways: a summary of the literature

**DOI:** 10.1108/JHOM-05-2020-0192

**Published:** 2021-03-24

**Authors:** Syaribah Noor Brice, Paul Harper, Tom Crosby, Daniel Gartner, Edilson Arruda, Tracey England, Emma Aspland, Kieran Foley

**Affiliations:** School of Mathematics , Cardiff University , Cardiff, UK; Velindre Cancer Centre , Cardiff, UK; Department of Decision Analytics and Risk, Southampton Business School , University of Southampton , Southampton, UK; Alberto Luiz Coimbra Institute-Graduate School and Research in Engineering , Federal University of Rio de Janeiro , Rio de Janeiro, Brazil

**Keywords:** Qualitative mapping, Cancer care pathways, Diagnostics, Treatment, Access barriers, Literature review

## Abstract

**Purpose:**

The study aims to summarise the literature on cancer care pathways at the diagnostic and treatment phases. The objectives are to find factors influencing the delivery of cancer care pathways; to highlight any interrelating factors; to find gaps in the literature concerning areas of research; to summarise the strategies and recommendations implemented in the studies.

**Design/methodology/approach:**

The study used a qualitative approach and developed a causal loop diagram to summarise the current literature on cancer care pathways, from screening and diagnosis to treatment. A total of 46 papers was finally included in the analysis, which highlights the recurring themes in the literature.

**Findings:**

The study highlights the myriad areas of research applied to cancer care pathways. Factors influencing the delivery of cancer care pathways were classified into different albeit interrelated themes. These include access barriers to care, hospital emergency admissions, fast track diagnostics, delay in diagnosis, waiting time to treatment and strategies to increase system efficiency.

**Originality/value:**

As far as the authors know, this is the first study to present a visual representation of the complex relationship between factors influencing the delivery of cancer care pathways.

## 1. Introduction

A care pathway can be defined as a general clinical plan that maps the trajectory of the patient through the healthcare system ( Altini *et al.* , 2020 ; Schrijvers *et al.* , 2012 ). As such, it involves many activities and can be seen as a complex adaptive system that responds to medical decisions, patient outcomes and local characteristics, among other factors. Indeed, care pathways are subjected to a complex interplay of factors that can be captured, for example using causal loop diagrams ( Littlejohns *et al.* , 2018 ; Sterman, 2000 ).

The importance of proper management and a clear understanding of the impact of care pathways in the quality of care is acknowledged in the literature ( Allen *et al.* , 2019 ). It is often argued that the input of patients, as well as healthcare specialists, is essential in the design of effective care pathways ( Donetto *et al.* , 2019 ). Whilst the design of care pathways to provide better patient experience and improved outcomes is an important area in the literature ( Currie and Harvey, 2000 ; Lawson *et al.* , 2006 ), this paper focuses on the analysis of the delivery of cancer care pathways to understand the complex interaction of factors that ultimately affect the quality of the service delivered to the patient.

Cancer is regarded as the second leading cause of death worldwide and accounts for approximately one in every six deaths around the globe ( World Health Organization, 2018 ). It is estimated that half of the UK population will develop cancer in their lifetime ( Queen Mary University of London, 2017 ).

Problems related to the availability and delivery of cancer care are common ( Prager *et al.* , 2018 ). The issues include the accessibility of cancer care services such as screening, diagnosis, treatment and the unpredictability in the delivery of these services ( Wang and Onega, 2015 ). Several countries have developed standardised pathways for cancer patients to address the latest issue and improve patient outcomes. Descriptions of standardised lung cancer pathways in Australia, the UK and Canada can be found in Department of Health and Human Services (2016) ; Lung Clinical Expert Group (2017) ; Cancer Care Ontario (2019) , respectively. Elsewhere, standardised pathways for cancer care have been found to correlate with improved survival rates for patients with seven distinct types of cancer in Denmark ( Jensen *et al.* , 2017 ). The pathways also contributed to the reduction of the diagnostic interval, i.e. the time elapsed from initial presentation to final diagnosis ( Weller *et al.* , 2012 ). Some studies have associated the implementation of standardised cancer pathways with increased efficiency, improved outcomes and higher patient satisfaction levels (e.g. Delilovic *et al.* , 2019 ; Gesme and Wiseman, 2011 ). However, negative effects have also been identified, such as longer waiting times for patients competing for the same resources (e.g. Delilovic *et al.* , 2019 ). This suggests that research on the implementation of standardised pathways that use shared resources should also consider the different patient groups sharing the resources.

Patient profiles may vary, and even the experiences of similar patients may be significantly different. Generally, variations may be due to the underlying heterogeneity of a patient's physical health and behaviours, professional uncertainty, external constraints, or diffusion of new knowledge and practices ( Alzahouri *et al.* , 2008 ). For example, similar patients may undertake distinct diagnostic procedures ( Alzahouri *et al.* , 2008 ); or the indications and the choice of emergency surgery procedures may vary according to local conditions ( Bosscher *et al.* , 2015 ). These uncertainties may further complicate an already involved decision-making process, but one way to mitigate their influence is by designing tools and patient pathways that benefit from multidisciplinary team discussions ( Bosscher *et al.* , 2015 ).

A report from the Organisation for European Cancer Institutes Accreditation and Designation program suggested that different cancer centres tend to have different numbers of pathologies with dedicated clinical pathways ( Saghatchian *et al.* , 2014 ). The variation also exists in the implementation of pathways with waiting time targets. In the UK, the National Health Service (NHS) guidelines established maximum waiting times of 2 weeks from referral to outpatient appointment and 62 days from referral to first treatment ( Department of Health, 2007 ).

Literature review studies have explored factors relating to cancer care delivery, albeit with different focuses. The effect of case management in cancer care could not be ascertained due to the scarcity of literature and variations in methodology ( Wulff *et al.* , 2008 ). On the other hand, delays due to both the practitioner and the patient have been correlated with similar risk factors, such as demographic, socio-economic, education and health conditions ( Macleod *et al.* , 2009 ). In addition, the lack of knowledge about cancer symptoms and the benefits of treatment were found to influence the delays in accessing the service ( Akuoko *et al.* , 2017 ; Jones *et al.* , 2014 ; Williams *et al.* , 2019 ).

The aforementioned literature studies have investigated factors related to either delay in the presentation or diagnosis and treatment. In contrast, this study aims to summarise the literature on the delivery of cancer care pathways, covering the studies from presentation to diagnosis and treatment. The objectives include investigating the factors associated with the timely and effective delivery of the cancer pathways, highlighting any interrelating factors, summarising the implemented strategies for delivering cancer pathways and reporting gaps in the literature.

## 2. Methodology

The literature search was conducted in March 2020 and covered SCOPUS, Science Direct, MEDLINE, PubMed, Web of Science, and PMC. The search used the following keywords: cancer* AND (“diagnostic pathway*” OR “patient pathway*” OR “care pathway*” OR “critical pathway*” OR “care map*” OR “clinical pathway*”). We constrained ourselves to studies written in English in the last 20 years. We excluded studies that covered topics related to testing devices, development of tools, genetic testing, tumour growth and surgical procedures. The quality of the papers was not considered. The selection process was administered by the lead author. The result was presented, discussed and disseminated to the team members who gave suggestions and contributed to the writing of the paper.

**Figure 1:**
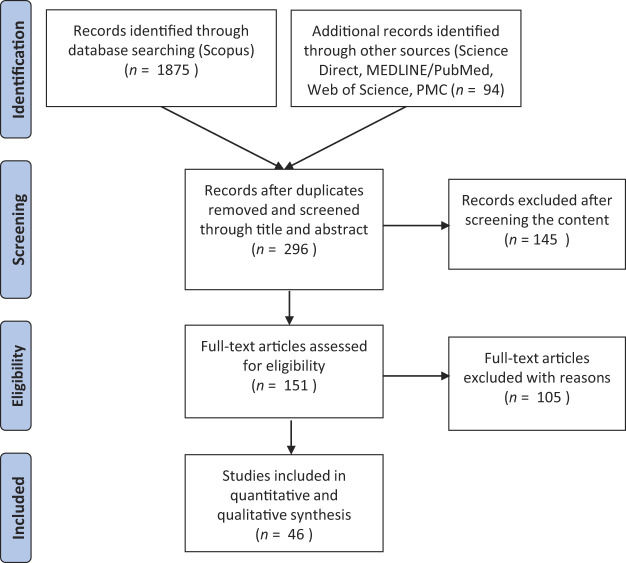
The selection process flow using simplified PRISMA diagram

Figure 1 summarises the selection process. The first filter identified 1,969 potential studies. This number reduced to 296 following the removal of duplicates and screening based on titles and abstracts. It was further trimmed to 151 after excluding content that did not comply with the inclusion criteria. Finally, 105 studies were excluded due to reasons such as being related to palliative or supportive care and not concerning the delivery of cancer pathways. This resulted in the selection of 46 papers for further analysis.

### 2.1 Causal loop diagram for cancer care pathways

Causal loop diagrams ( Sterman, 2000 ) are a system's dynamics tool developed to convey the interplay of factors in complex social systems by promoting a holistic view of the problem at hand. The rationale is to develop a diagram that conveys the existing relationships between pairs of factors to unveil a complete picture of the system to stakeholders and decision-makers.

The present study develops a causal loop diagram to highlight the causal link or relationship between each pair of factors/topics (also dubbed *variables* ) investigated in the studies. In order to simplify the analysis whilst also retaining the essence of the articles, we make use of the topics that appear more frequently across all studies. The links between pairs of factors (variables) are represented by arrows in the resulting diagram. The arrows may be non-directional, when the relationship exists but no causality is attributed. Another type of arrow is uni-directional when the direction of causality is established. This type of arrow will have polarity (i.e. an established direction). The diagram does not capture any bi-directional relationship, since such a type of relationship was not found in the studies. It is important to note that the use of the tool is to facilitate the description of relationships between factors. These may not necessarily represent causality.

The factors were selected from the surveyed studies, as well as their pairwise relationships. For the sake of illustration, consider for example a study by Hansen *et al* . (2008) that investigated factors related to delay in cancer diagnosis. The study found that patients' socio-economic situation and age correlated with the delay in cancer diagnosis. Based on these results, the socio-economic and age factors were linked to patient delay in the diagram, see Figure 9 .

The causal loop diagram was built using the software Vensim PLE, version 7.3.5 from Ventana Systems Inc ( https://vensim.com ). Individual analyses of specific themes were also carried out to describe pertinent variables following the surveyed literature.

## 3. Results

**Figure 2:**
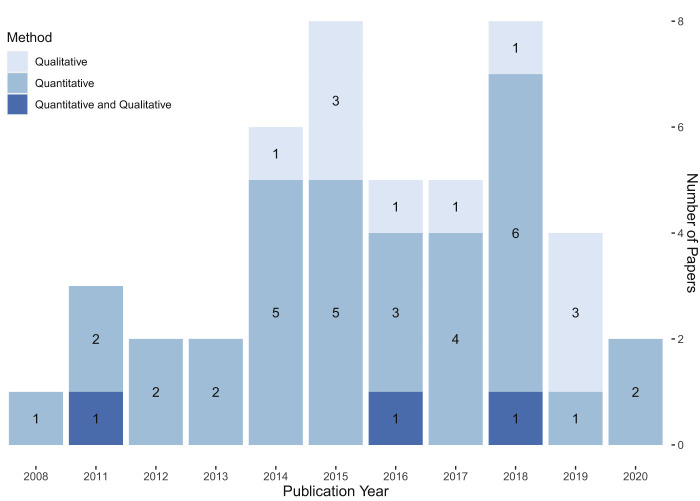
Literature classification by year of publication and research methods

Figure 2 highlights the number of works published yearly from 2008 to 2020. It displays an upward tendency in the number of yearly publications from 2012 to 2018, which might indicate an increasing interest in the subject. Interestingly, no publications were found in 2009 and 2010, which is not to say that no research was conducted in that period. Overall, quantitative research was more prevalent than qualitative research.

**Figure 3:**
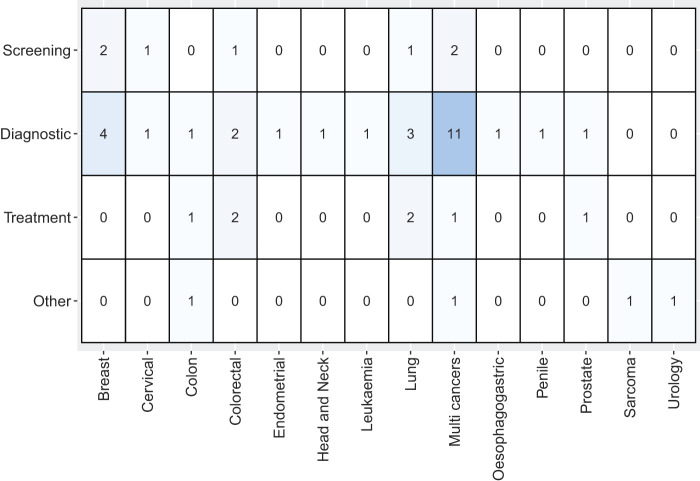
Distribution of cancer types by different care services

**Figure 4:**
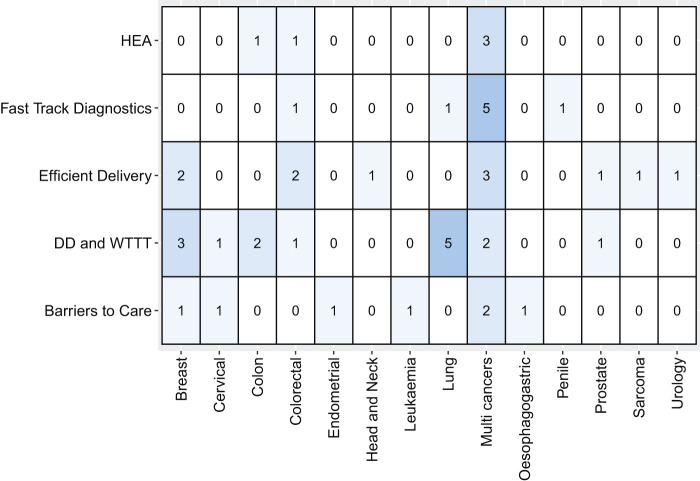
Distribution of themes found in the literature. HEA (hospital emergency admission), DD and WTTT (delayed diagnosis and waiting time to treatment)

Figure 3 presents the classification of the literature based on the types of services and cancers found in the literature. The number of papers dealing with “Diagnostics” stands out ( *n* = 27), followed by studies concerned with “Treatment” and “Screening” ( *n* = 7 and *n* = 6, respectively). The category “Other” included studies related to efficiency and emergency admissions ( *n* = 4). Studies on diagnostics mainly involved multiple types of cancer. We identified five emerging and important themes related to cancer care services, namely hospital emergency admissions, fast track diagnostics, efficient delivery of cancer care services, delay in diagnosis and waiting time to treatment, and barriers to care. Figure 4 shows the distribution of these themes according to the cancer specialties. Delay in diagnosis and waiting time to treatment is the most common theme in the surveyed studies ( *n* = 15), followed by efficient delivery of care services ( *n* = 10). The next most frequent theme is fast track diagnostics ( *n* = 8), followed by access barriers to care ( *n* = 7). Studies on fast track diagnostics mainly covered multiple cancer types ( *n* = 5). Finally, the majority of lung cancer studies explored issues related to delay in diagnosis and waiting time to treatment ( *n* = 5).

The derived causal loop diagrams feature emerging themes. Further analyses describe the relations between factors in the form of causal trees. Each subsequent subsection conveys a causes tree related to a specific theme and further discusses the theme. The theme *delayed diagnosis and waiting time to treatment* is further split into *delay in diagnosis* , and *treatment delay* . Given the complexity of the problem, each individual causes tree may fail to capture the intricate connections among variables, as there may be common factors that influence multiple themes. However, the discussion will be general enough to cover other parts of the diagram related to the theme.

To complement the analysis concerning individual themes, Appendix features a comprehensive picture of the system as a whole in the form of a causal loop diagram. It conveys the relationship among distinct influences of different themes, as well as the connections between the themes in terms of common influences. Even though we cannot claim that the resulting diagram is a complete picture of reality, it depicts the perceptions of the surveyed studies regarding the complex issues connected to the delivery of cancer care.

### 3.1 Access barriers to care

**Figure 5:**
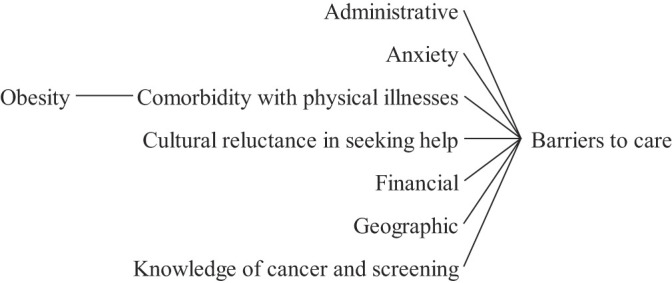
Factors influencing access barriers to cancer care

Figure 5 presents a causes tree highlighting factors that can compromise the access to cancer care services. Administrative barriers include poor communication and service configuration, as well as the lack of a uniform service standard for all patients ( Cusimano *et al* ., 2019 ). Anxiety may result from a poor relationship between doctor and patient ( Clarke *et al* ., 2014 ), from the natural apprehension regarding a possible cancer diagnosis ( Huddy *et al* ., 2016 ), or the increased tension due to a delayed diagnosis ( Nessim *et al* ., 2015 ).

Obesity and other co-morbidities may affect the access of patients to the point of care and produce delays in the diagnosis ( Guldbrandt *et al* ., 2015 ). Obesity in particular has been associated with stigma and poor communication on the part of the service provider ( Cusimano *et al* ., 2019 ). The cultural reluctance in seeking help contributes to delay in diagnosis and treatment, which may lead to patients presenting with late-stage cancer ( Huddy *et al* ., 2016 ).

Concerning financial aspects, patients from deprived areas have been found more prone to late-stage diagnosis ( Maclean *et al* ., 2015 ) and more likely to require emergency care ( Maringe *et al* ., 2018 ). Somewhat correlated are geographical factors, such as the distance to the available cancer centres ( Turner *et al* ., 2017 ), the area of residence of patients ( Maclean *et al* ., 2015 ), which is also correlated to deprivation indices ( Tin *et al* ., 2018 ; Tsang *et al* ., 2013 ) and the availability of diagnostic procedures and treatments in the vicinity ( Huddy *et al* ., 2016 ). Unsurprisingly, longer travel times to cancer centres and residing in a deprived area are associated with poorer patient outcomes.

The knowledge of patients regarding the cancer types, the treatments and the awareness concerning the benefits of early treatment, play an important role in the outcomes ( Redaniel *et al* ., 2015 ; Momberg *et al* ., 2017 ). Better informed patients tend to make better decisions regarding screening and diagnostic strategies, thereby improving early presentation indices. Finally, the understanding and trust underlying the patient-doctor relationship are also important to accelerate diagnosis and thereby improve outcomes, particularly in paediatric cancers ( Clarke *et al* ., 2014 ).

### 3.2 Efficient delivery of care pathways

**Figure 6:**
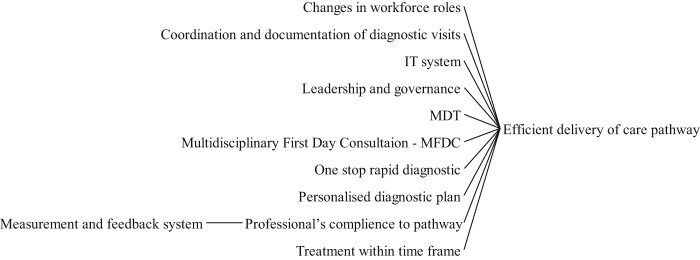
Factors influencing efficiency in delivering cancer care

Figure 6 summarises strategies proposed and implemented to deliver improved and faster pathways. Generally, developing a cancer pathway involves prescribing performance measures and planning in accordance with these measures for post implementation and monitoring. Efficient delivery, in turn, demands leadership, coordination, information technology systems and governance ( Pitter *et al* ., 2019 ).

Multidisciplinary teams have a leading role in cancer care delivery. Because of this importance, multidisciplinary team meetings should be held frequently to avoid delays in diagnosis ( Redaniel *et al* ., 2015 ; Van Huizen *et al* ., 2018 ). The literature suggests that a well-defined hierarchy can contribute to speed up treatment decisions and develop automated decisions for simple cases ( Lamb *et al* ., 2014 ; Redaniel *et al* ., 2015 ). For rare cancer types, information technology can help identify courses of treatment when specialists are scarce ( Kasper *et al* ., 2018 ).

Rapid one-stop pathways have also been demonstrated effective for certain types of cancer ( Bass *et al* ., 2018 ; Haddow *et al* ., 2016 ), and cost-effective in a more generalist setting ( Sewell *et al* ., 2020 ). Also essential for the delivery of a care pathway is the existence of early intervention or screening programs ( Cariou *et al* ., 2018 ) and the proper definition and application of standardised pathways ( Hoverman *et al* ., 2011 ; Kubal *et al* ., 2016 ; Quan *et al* ., 2012 ). This is to ensure that personalised diagnostic plans are coherent and independent of the team overseeing the pathway, and that the best experiences are shared with management and fellow specialists.

Finally, effective delivery of care requires administrative support that ensures seamless access to associated services such as psychological assistance ( Franchi *et al* ., 2013 ) and rehabilitation services ( Stout *et al* ., 2019 ). The administrative support may be a part of the cancer service which acts as a bridge between the cancer service and the associated service that is required. These supportive services may not have direct impact on the delivery of cancer pathways, nonetheless they provide support which may improve patients' experience.

### 3.3 Hospital emergency admission

**Figure 7:**
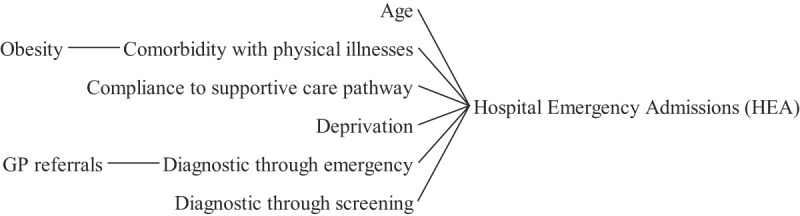
Factors influencing hospital emergency admission

Figure 7 depicts the main factors that contribute to hospital emergency admissions, according to the reviewed literature. These include age, deprivation, and comorbidity with other physical illnesses ( Kreys *et al* ., 2014 ; Maringe *et al* ., 2018 ; Tsang *et al* ., 2013 ).

Emergency visits can follow a GP referral ( Black *et al* ., 2015 ; Guldbrandt *et al* ., 2015 ) or be completely unplanned ( Ortiz-Ortiz *et al* ., 2016 ). In the context of cancer pathways, unplanned emergency visits are in general a symptom of a failure in the proper delivery of care that results, for example, in late-stage diagnosis. Hence, proper prevention and screening policies ( Cariou *et al* ., 2018 ) as well as improved cancer awareness in primary care can help mitigate hospital emergency admissions in cancer care ( Kreys *et al* ., 2014 ; Tsang *et al* ., 2013 ), provided that proper care is available for the patient ( Turner *et al* ., 2017 ).

### 3.4 Fast track diagnostics

**Figure 8:**
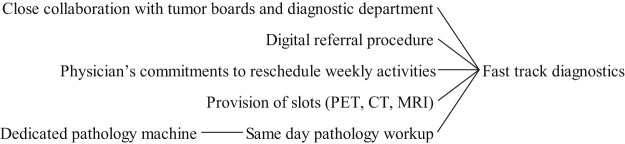
Factors influencing fast track strategy

Figure 8 summarises the factors associated with the implementation of fast track cancer pathways. Reported benefits of fast track programs include shorter diagnostic intervals and faster access to first treatment, as well as standardised protocols for a referral to secondary care ( Guldbrandt *et al* ., 2015 ; Jensen *et al.* , 2014 ; Prades *et al* ., 2011 ; Sewell *et al* ., 2020 ). However, the results reported in the literature are often myopic, for they fail to consider the additional burdens imposed on non-fast-track patients. Unfortunately, the latter group is comprised of the majority of patients ( Zhou *et al* ., 2018 ).

Fast track services often resort to dedicated resources and prioritised use of installed capacity which intuitively lead to better delivery of care for prioritised patients ( Jakobsen and Jensen, 2016 ; Van Harten *et al* ., 2018 ). However, it is important to contrast such an improvement with the eventual degradation of the delivery of care for non-prioritised patients.

### 3.5 Delay in diagnosis and waiting time to treatment

**Figure 9:**
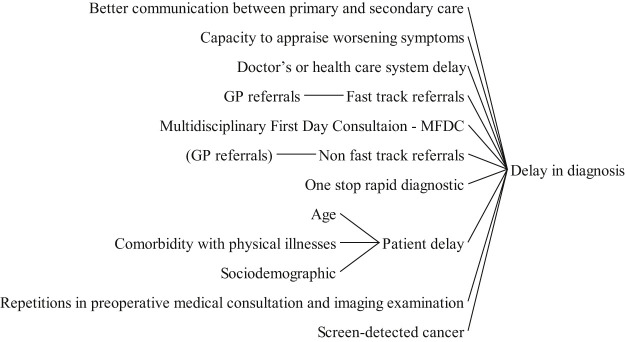
Factors influencing delay in diagnosis

**Figure 10:**
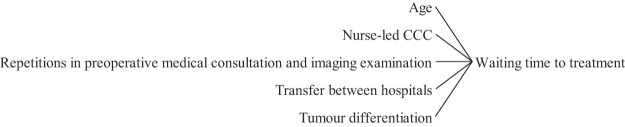
Factors influencing waiting time to treatment

Figure 9 highlights the contributing factors to the delay in diagnosis. As expected, there are some overlaps with the factors that lead to efficient delivery, which would be expected to prevent unnecessary delays. Figure 10 highlights the main issues that contribute to the pre-treatment and post-diagnostic delays, according to the surveyed literature.

Early diagnosis and treatment are of the utmost importance in cancer care, hence it is no surprise that time to first treatment and time to diagnosis are among the performance functions evaluated in cancer care ( Black *et al* ., 2015 ; Nessim *et al* ., 2015 ). Delayed diagnosis can be due to administrative and systemic issues, primary care delivery, or patient related issues ( Hansen *et al* ., 2011 ). Patient delays are correlated to socio-demographic characteristics, such as gender, awareness of cancer, economic status, alcohol intake and tobacco consumption ( Hansen *et al* ., 2008 ; Huddy *et al.* , 2016 ; Lim *et al* ., 2014 ).

System delay may stem from unnecessary or delayed diagnostic procedures ( Chiarelli *et al* ., 2017 ; Laerum *et al* ., 2020 ; Héquet *et al* ., 2017 ) but also from a tendency of admitting late-stage patients in the system ( Forrest *et al* ., 2014 ; Nessim *et al* ., 2015 ; Redaniel *et al* ., 2013 ). Unsurprisingly, better communication between primary and secondary care, and better qualified personnel can help mitigate system delay ( Black *et al* ., 2015 ). Administrative issues include mismanagement of patient transfers between services and levels of care ( Iachina *et al* ., 2017 ), as well as inadequate management of referral, consultation and booking for treatment ( Stokstad *et al* ., 2019 ; Wasserman *et al* ., 2015 ).

A central nurse-led coordination of cancer care has been associated with improved outcomes and better delivery of cancer services ( Aarhus *et al* ., 2019 ; Blakely *et al* ., 2015 ; Wulff *et al* ., 2012 ). One possible reason for this is the proximity and empathy between nurses and patients ( Tod *et al* ., 2015 ).

## 4. Discussion

The study has identified literature pertaining to factors as well as strategies associated with the delivery of cancer care pathways from presentation to diagnosis and treatment. The analysis using qualitative mapping, in this case a causal loop diagram, revealed that factors and strategies are interlinked. It highlighted the complexity of the cancer care pathways in general, and that factors influencing a certain part of the cancer care pathway may also affect other areas in the pathway.

Access barriers to care are acknowledged in many passages of the surveyed literature, and the issues that lead to such barriers are important to both primary and secondary care. Studies that investigated access barriers sometimes have done so from the perspective of individual patients and other times have contemplated the perspective of the service providers. Factors associated with access barriers, from the patient perspective, include socio-economic status, demographic profile or comorbidity with other physical health conditions. These support previous reviews, such as Macleod *et al.* (2009) and Williams *et al.* (2019) . The literature showed that these factors are not only associated with access to primary care but also with hospital emergency access ( Tsang *et al* ., 2013 ) or even hospital admissions in general.

Lack of knowledge about a certain cancer program available in the community or regarding cancer itself is a recurring theme in the literature. Such knowledge is invaluable not only for the population at risk but also for health professionals ( Williams *et al.* , 2019 ). It is not difficult to see that a lack of understanding about the benefits of screening programs for certain types of cancer, or about the associated diagnostic procedures, can affect the decision of individual patients on whether or not to join screening initiatives ( Momberg *et al* ., 2017 ). In a certain ethnicity, the lack of knowledge and awareness of cancer can be found in both developed countries ( Jones *et al.* , 2014 ) and developing countries ( Akuoko *et al.* , 2017 ).

Studies have identified a system of healthcare factors associated with the delivery of the cancer care pathway. In general, these might relate to resources such as availability of certain diagnostic procedures or treatments, the availability of experts in rare cancers, the management of diagnosis and treatment pathways, the communication between health professionals and the patients, and between service providers. These factors highlight gaps in accessing quality care in some European countries ( Kasper *et al* ., 2018 ). Multidisciplinary support via multidisciplinary team meetings play an important role in cancer care delivery ( Lamb *et al* ., 2014 ; Van Huizen *et al* ., 2018 ). The effectiveness of the multidisciplinary teams depends upon the implemented strategies. These include prioritising cases based on the type and condition of the tumour and making decisions for simple cases based on a standardised cancer pathway ( Lamb *et al* ., 2014 ).

Patient related factors together with the healthcare system contributed to delays in diagnosis and receiving first cancer treatment ( Hansen *et al* ., 2011 ; Huddy *et al.* , 2016 ). Raising cancer awareness in the community, and better coordination and communication between service providers are amongst suggested strategies to mitigate the delays ( Black *et al* ., 2015 ).

The study did not limit the delivery of care pathways for specific cancers or services. The results showed that at a high-level abstraction, factors influencing delivery at a certain phase of cancer care might be similar regardless of the cancer types. The analyses give insights into the complex care pathways, capturing not only primary care but also secondary and even tertiary care. As a result, the study presented a preliminary model toward a comprehensive description of factors and strategies influencing cancer care processes. Such a model, which provides a comprehensive knowledge regarding the cancer care pathways, may support the decision making process ( Butler *et al* ., 2013 ).

Finally, an apparent gap in the literature is the lack of studies analysing the holistic effects of cancer pathways in the health system ( Zhou *et al.* , 2018 ). In particular, the literature lacks studies evaluating the decrease in the quality of service for patients competing for the same resources when cancer care is prioritised.

### 4.1 Strategies and recommendations

Although some findings in the literature are limited to a given setting, this section exploits recurring conclusions that can be used to inform policy making in general and help optimise cancer care pathways. Firstly, it has been found that cancer awareness is positively correlated with improved outcomes and early presentation. Hence, we recommend developing policies to raise cancer awareness in the community, as well as continuous training and information exchange with healthcare professionals in primary care. Early intervention and screening plans are also very important to ensure early presentation. The intervention should be tailored to the context and address an individual's issues related to access barriers ( Detterbeck *et al* ., 2013 ).

Multidisciplinary teams and discussion boards should also be included in the pathways to improve outcomes and recommendations. However, these discussions should be frequent enough to prevent these meetings from becoming a bottleneck that delays diagnosis. The multidisciplinary teams should establish automated decisions for simpler cases to speed up diagnosis. This may be supported by extending the role of the cancer nurse specialists in the multidisciplinary teams.

Pathways should be standardised to ensure that diagnostic plans are independent of the team overseeing the pathway, and information exchange should guarantee that the best experiences are shared with management and fellow specialists. The pathways should provide a comprehensive cancer care program, be implemented, and subject to regular update ( Christensen *et al* ., 2017 ). In addition, proper information technology (IT) support and rapid access to associated services such as psychological assistance and rehabilitation services should be ensured to improve outcomes.

Finally, fast track diagnostics should be considered to speed up treatment decisions. However, the planning of a fast track service should consider the impact to all patients that make use of the shared resource that would be prioritised, to make sure that the overall effect is positive. To avoid delays, preemptive measures should also be considered to prevent delays related to socio-economic and demographic characteristics. Finally, a channel should be established between primary and secondary care, as such a channel has been associated with delay mitigation ( Brown *et al* ., 2014 ).

### 4.2 Study limitation

Due to the keywords and inclusion criteria used for the literature search, the study may not have covered the literature in its entirety. The search was limited to articles published in journals. Future studies may update this effort and include grey literature such as policy and organisational reports.

The resultant causal loop diagram model was developed using combined factors and strategies discussed in the included studies. In addition, the quality of each study was not assessed and has not been taken into account in the analysis. Hence, the resulting diagram might capture the subjectivity of the authors in summarizing the results, as well as biases found in the studies. Further research might contest and refine the model by including more evidence from studies. Others might take some ideas presented in the model and turn them into a quantitative model that can be used to investigate the interrelationship between the factors. Such a quantitative model might capture not only the patient flow in the cancer care system but also the factors influencing the flow.

The study is limited to cancer care pathways in relation to diagnostic and treatment delivery. Factors related to patient outcomes such as survival and quality of life were not included. Future studies may include literature discussing patient outcomes in relation to cancer care pathways. The inclusion of such literature may highlight the important links between other support care pathways and the diagnostic and treatment care pathways. This would be a step towards capturing a holistic view of healthcare systems in cancer care.

## 5. Conclusion

Factors influencing the delivery of cancer pathways are myriad and complex. In general, the factors may relate to the individual patient or the system of care. Factors such as patient characteristics, socio-economic conditions, knowledge of cancer and cancer symptoms are interrelated and influence different cancer services. The results not only highlighted the factors associated with delay in diagnosis or treatment, but also the strategies proposed in the literature to deliver timely cancer pathways, such as fast track diagnostics.

The successful delivery of cancer pathways was supported by factors such as IT and information systems, multidisciplinary teams, and case management, among others. However, the number of studies found is not large, especially with respect to specific cancer types. More studies are needed on the successful delivery of cancer pathways, particularly focusing on specific cancer types, as different cancers require distinct cancer pathways.

This study provided, by means of a causal loop diagram, a comprehensive picture of the factors influencing cancer care. The resulting model developed in the study can be regarded as a preliminary model representing complex cancer care pathways. Future studies should confirm or mitigate the links between factors by including up-to-date evidence. The findings give rise to recommendations and insights that can guide practitioners in the development of new and the refinement of existing cancer care pathways.

## Figures and Tables

**Figure A1:**
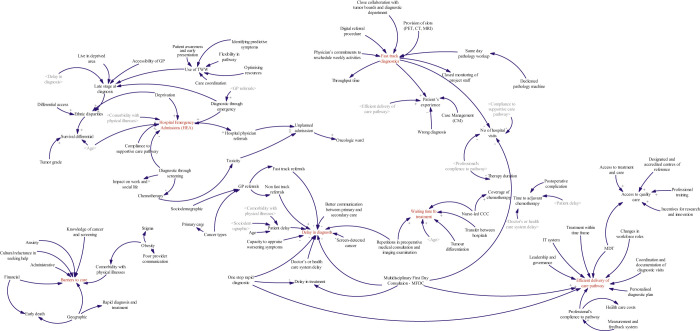
CLD Diagram for factors influencing the delivery of cancer pathways, developed using Vensim PLE
